# Comparison of LIRADS 5 Image Guided Core Biopsy Derived From Formalin Fixed and Frozen Tissue Cores for Radiogenomics and Radioproteomics Analysis in Well, Moderate and Poorly Differentiated Hepatocellular Carcinoma

**Published:** 2024-04-22

**Authors:** Margaret Simonian, David S.K. Lu, Julian Whitelegge, Whitaker Cohn, Preeti Ahuja, William Hsu, Steven S. Raman

**Affiliations:** 1 Dept of Radiological Sciences, David Geffen School of Medicine, UCLA, USA; 2 Semel Institute for Neuroscience & Human Behavior, Pasarow mass spectrometry lab, David Geffen School of Medicine, UCLA, USA

**Keywords:** Liver HCC, radioproteomics, radiogenomics, biomarkers

## Abstract

The aim of this pilot study is to evaluate and compare the quality of the genomics and proteomics data obtained from paired Formalin Fixed Paraffin Embedded (FFPE) and frozen (FF) tissue percutaneous core biopsies of Liver Imaging Reporting and Data System 5 (LIRADS 5) hepatocellular carcinoma (HCC) of varying histological grades. The preliminary data identified differentially expressed proteins and genes in poor, moderate and well differentiated HCC biopsies, with a greater efficacy in fresh frozen samples. The data offered valuable insights into the characteristics and suitability of samples for future studies.

## Introduction

Hepatocellular Carcinoma (HCC) is the most common liver cancer and it’s the leading cause of cancer-related deaths worldwide. HCC patients do not respond to most systemic therapies [1,2].

Current diagnostic tests are blood, imaging, and biopsy, with no useful predictive biomarkers. Very few proteomics biomarkers have been studied for liver HCC and their correlation to clinical behavior and response to therapy is limited [3–5]. Targeted therapies may prolong survival in a minority of patients but are not personalized [6,7]. Therefore, we aim to analyze the tissue biopsies of HCC patients of different grades, using proteomics and genomics analysis, and combine it with our imaging CT/MRI data (radiomics) to generate radioproteomics and radiogenomics data to better predict HCC subtypes and ultimately offer personalized medicine for HCC patients.

Most of percutaneous image guided clinical biopsies are stored as FFPE, which is the standard method used by pathologists to diagnose histologically HCC grades. However, comparison studies of other type of cancer tissues, suggested that using FF tissues have advantages over FFPE, such as: proteins/ DNA/ RNA are better preserved, the variability if FF tissues are lower than FFPE tissues which can affect the data quality, and FF samples can be stored for more than 2 years with no risk of DNA/protein degradation unlike FFPE samples [8,9].

Here we report the preliminary data that we obtained by comparing three paired FFPE and FF tissue biopsies, derived from 18 G percutaneous biopsy of LIRADS 5, three histological grades (well differentiated, moderate differentiated, and poorly differentiated) HCC, to determine the most optimal tissue type for our larger study.

## Materials and Methods

### RNA-seq library construction and sequencing

RNA-seq libraries of three paired FFPE and FF tissue biopsies of three histological grades were prepared with KAPA mRNA HyperPrep Kit with RiboErase (Roche). rRNA was depleted by hybridization of complementary DNA oligonucleotides, followed by treatment with RNase H and DNase. The first strand cDNA synthesized using random priming followed by second strand synthesis converting cDNA:RNA hybrid to double-stranded cDNA (dscDNA), and incorporating dUTP into the second cDNA strand. cDNA generation is followed by end repair to generate blunt ends, A-tailing, adaptor ligation and PCR amplification.

Sequencing was performed on Illumina NovaSeq6000 for a paired end 2x50 run. Data quality check was done on Illumina SAV. Demultiplexing was performed with Illumina software. The reads were mapped by STAR 2.7.9a [10] and read counts per gene were quantified using the human genome GRCh38.104. In Partek Flow [9], read counts were normalized by CPM +1.0E-4. Differential expression of genes was measured using the gene set enrichment (GSA) algorithm in Partek Flow, generating unfiltered as well as filtered datasets. Statistical filters for differential expression were set at fold-change > 2 and p < 0.01.

### Deparaffinization of FFPE Tissues

FFPE tissue scrolls were placed in Eppendorf tubes, 1 mL of 100% xylene was added for 10 minutes to deparaffinized the tissue scrolls. Centrifuged 3 times, at 16,000 x g for 3 min, supernatant was discarded. Followed by 3 mL of 100% ethanol, for 3 min, pelleted at 16,000 x g for 3 minutes. This step was repeated an additional two times. Supernatant was discarded.

### Protein digestion and TMT labelling

Fresh frozen and FFPE tissue homogenization was carried out using 12 mM sodium lauryl sarcosine, 0.5% sodium deoxycholate, and 50 mM triethyl ammonium bicarbonate TEAB, in ultrasonic cell disruptor for 20 seconds. Samples were then centrifuged at 16,000 × g for 5 min, supernatant was collected, heated at 95°C for 1 hour and sonicated for 5 minutes. The total protein concentration of the samples was determined using BCA Protein Assay Kit (Pierce, Thermo Fischer Scientific). The standard curve was generated using Bovine serum albumin. The samples were treated with tris (2-carboxyethyl) phosphine (10 μL, 55 mM in 50 mM TEAB, 30 min, 37 °C), followed by treatment with chloroacetamide (10 μL, 120 mM in 50 mM TEAB, 30 min, 25 °C in the dark). They were then diluted five-fold with aqueous 50 mM TEAB and incubated overnight with Sequencing Grade Modified Trypsin (1 μg in 10 μL of 50 mM TEAB; Promega, Cat # V511A, Madison, WI, USA), 1 μg of trypsin per sample used regardless of protein extraction yield. After digestion an equal amounts of peptides TMT were labelled and combined for MS analysis, modified protocol from Simonian, et al. [11]. An equal volume of ethyl acetate/trifluoroacetic acid (TFA, 100/1, v/v) was then added, followed by avigorous mix (5 min) and centrifugation (13,000 × g, 5 min). The supernatants were discarded, and the lower phases were dried in a centrifugal vacuum concentrator. The samples were then desalted using a modified version of Rappsilber’s, et al. protocol [12], in which the dried samples were reconstituted in acetonitrile/water/TFA (solvent A, 100 μL, 2/98/0.1, v/v/v) and then loaded onto a small portion of a C18-silica disk (3M, Maplewood, MN, USA) placed in a 200 μL pipette tip. Prior to sample loading, the C18 disk was prepared by sequential treatment with methanol (20 μL), acetonitrile/water/TFA (solvent B, 20 μL, 80/20/0.1, v/v/v), and finally with solvent A (20 μL). After loading the sample, the disc was washed with solvent A (20 μL, eluent discarded) and eluted with solvent B (40 μL). The collected eluent was dried in a centrifugal vacuum concentrator. The samples were then chemically modified using a TMT11plex Isobaric Label Reagent Set (Thermo Fisher Scientific, Cat # A34808, Waltham, MA, USA) as per the manufacturer’s protocol. The TMT-labeled peptides were dried and reconstituted in solvent A (50 μL), and an aliquot (2 μL) was taken for measurement of total peptide concentration (Pierce Quantitative Colorimetric Peptide, Thermo Fisher Scientific, Waltham, MA, USA). The samples were then pooled and desalted again using the modified Rappsilber’s protocol.

### LC MS/MS

Peptides were injected onto a reverse phase nanobore HPLC column (AcuTech Scientific, C18, 1.8um particle size, 360 um x 20 cm, 150 um ID), equilibrated in solvent A (water/acetonitrile/FA, 98/2/0.1, v/v/v) and eluted (300 nL/min) with an increasing concentration of solvent B (acetonitrile/water/FA, 98/2/0.1, v/v/v: min/% F; 0/0, 5/3, 18/7, 74/12, 144/24, 153/27, 162/40, 164/80, 174/80, 176/0, 180/0) using an EASY-nLC II (Thermo Fisher Scientific). The effluent from the column was directed to a nanospray ionization source connected to a hybrid quadrupole-Orbitrap mass spectrometer (Q Exactive Plus, Thermo Fisher Scientific) acquiring mass spectra in a data-dependent mode alternating between a full scan (m/z 350-1700, Automated Gain Control (AGC) target 3 x 106, 50 ms maximum injection time, FWHM resolution 70,000 at m/z 200) and up to 15 MS/MS scans (quadrupole isolation of charge states 2-7, isolation window 0.7 m/z) with previously optimized fragmentation conditions (normalized collision energy of 32, dynamic exclusion of 30 s, AGC target 1 x 105, 100 ms maximum injection time, FWHM resolution 35,000 at m/z 200).

### Proteomics Data Analysis

Raw proteomic data were searched against a Uniprot database containing the complete reference human proteome (ID: UP000005640, Gene Count: 20597) using SEQUEST-HT (including dynamic modifications: oxidation (+15.995) on M, deamidation (+0.984) on N/Q, and carbamidomethyl (+57.021), phosphorylation (+79.966) on S/T/Y) in Proteome Discoverer (Version 2,4, Thermo Scientific), which provided measurements of relative abundance of the identified peptides. Decoy database searching was used to generate high confidence tryptic peptides (FDR < 1%). Tryptic peptides containing amino acid sequences unique to individual proteins were used to identify and provide relative quantification between different proteins in each sample.

## Results

**Figure 1.**
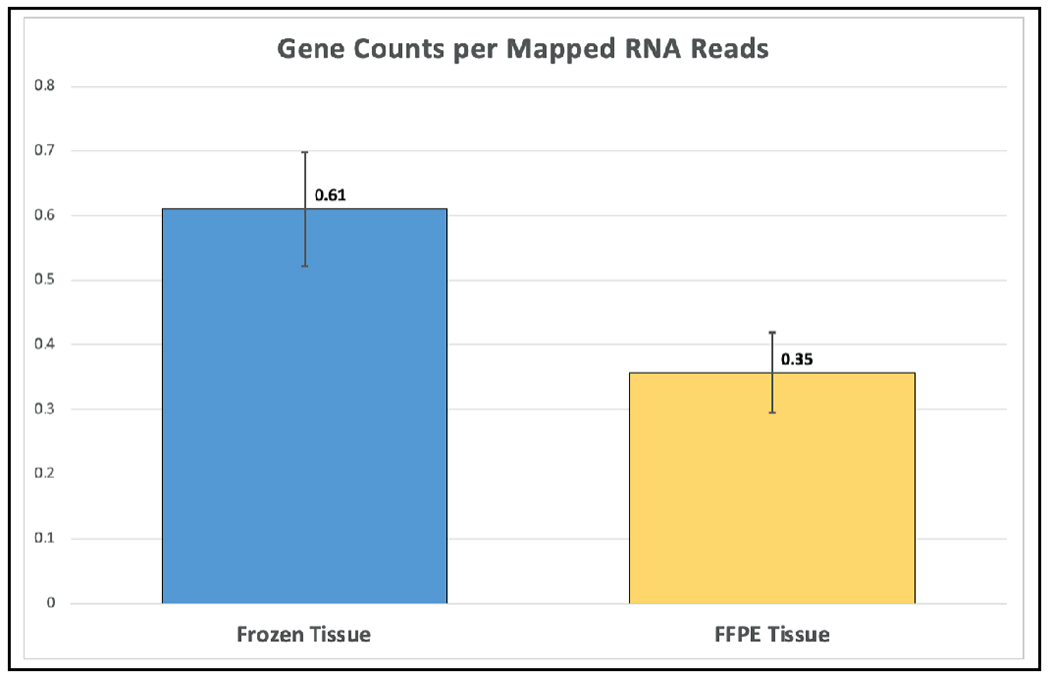
Higher gene counts in FF tissues over FFPE

**Figure 2.**
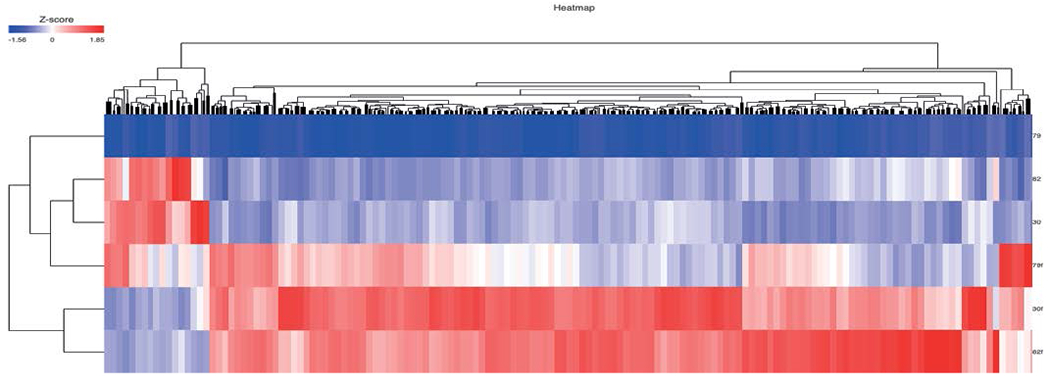
Heat map of overlapping genes in FFPE and FF in all phenotypes. (30 = FF moderate, 30f = FFPE moderate, 82 = FF well, 82f = FFPE well)

**Figure 3.**
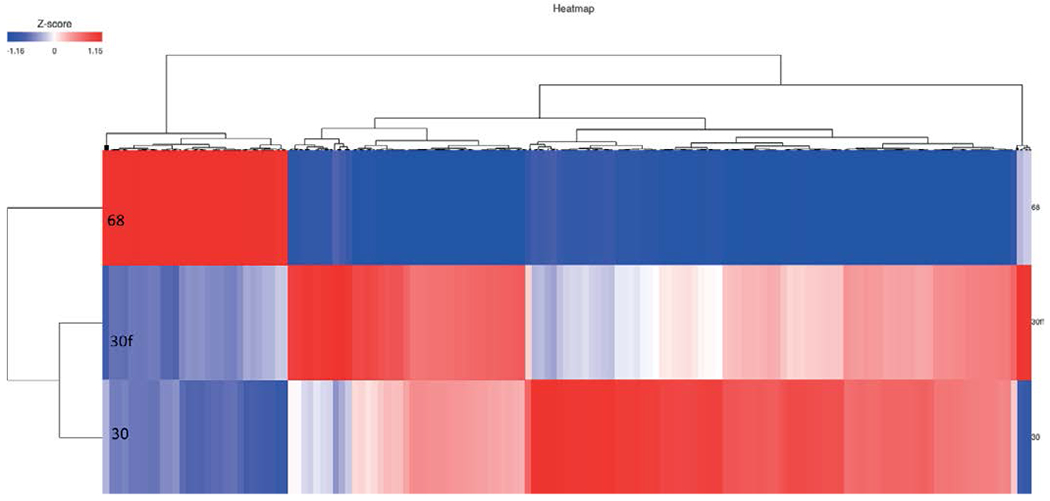
Heat map of moderate vs poor in FFPE and FF. (68 = poor, 30 = FF moderate, 30f = FFPE moderate)

RNA-seq identified 12,791 genes with 594 overlapping differentially expressed genes. The gene quantification efficiency of sequencing data gathered from FF was markedly higher than from FFPE tissues, with average gene counts per mapped reads of (0.61) vs (0.35) respectively (Figure 1). However, the overlapping upregulated genes in FFPE were higher than FF in the three histological grades, likely, due to tissue heat response associated with FFPE sample preparation. On histological grades comparison analysis, the overlapping upregulated genes in the moderately differentiated tissue cores were slightly higher than well differentiated tissue and markedly higher than poorly differentiated tissue cores in both FF and FFPE biopsy samples (Figures 2 and 3).

From the 594 overlapping genes, 5 genes were significantly upregulated (fold-change > 2) in moderate vs well differentiated tissue cores in both FF and FFPE, with greater fold change in FF samples e.g., MBL2 expression in (moderate FF) vs (well FF) = 25-fold, while in (moderate FFPE vs (well FFPE) = 3-fold; GLUL expression in (moderate FF) vs (well FF) = 27-fold, while in (moderate FFPE vs (well FFPE) = 5-fold (Table 1).

Additionally, 30 more genes were upregulated in moderate vs well differentiated in both FF and FFPE tissue cores, but with a fold-change < 2.

**Figure 4.**
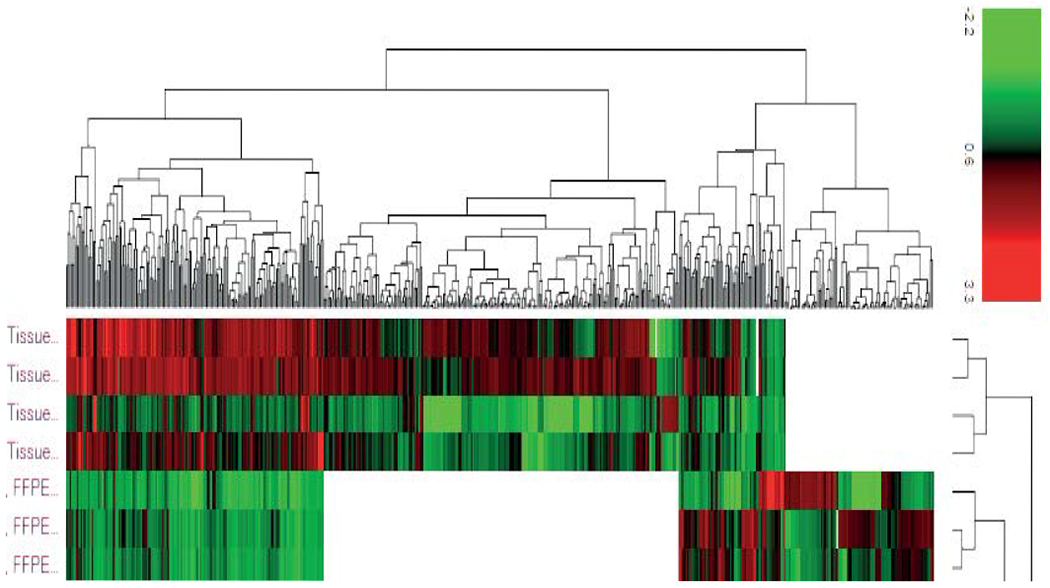
Heat map of all protein identified in FF and FFPE of liver HCC from proteomics analysis. The white patches indicates that proteins were not detected

The proteomics data identified 466 proteins in FF and 321 proteins in FFPE, with 222 overlapping differentially expressed proteins. More upregulated proteins were identified in FF vs FFPE is all phenotypes, including in the overlapping proteins, with greater fold-change in FF, due to higher concentration of proteins in Frozen tissue (Figure 4)

Within overlapping proteins comparison analysis, many proteins were upregulated in moderate vs well differentiated tissue cores, in both FF and FFPE, (209 and 190 respectively), with a greater fold change in FF (Supplementary Table 2).

Additionally, 195 proteins were upregulated in poor vs moderate differentiated tissues in FF, and 214 proteins were upregulated in poor vs well differentiated tissues in FF, some of proteins are presented in Table 2, more are in supplementary Table 2.

Many of the genes and proteins identified in this study play role in cancer progression, cell proliferation and immune response.

## Discussion / Conclusion

The proteomics data was in agreement with the RNA-Seq data. Both FF and FFPE can be used, with higher gene and protein quantification efficacy in the FF tissue cores.

This study revealed the relative strengths and limitations of percutaneous biopsy derived from 18 G percutaneous LIRADS 5 HCC of varying histological grades in FFPE and FF tissues for genomics and proteomics analysis, and offered valuable insights into the characteristics and suitability of samples. Understanding these aspects is crucial for making informed decisions in the planning and execution of future experiments.

While we recognize the importance of moving to proteoform analysis, the proteogenomic analytical approach used in this pilot study was only looking at correlations with RNA data. Our future study will focus on utilizing a larger number of FF biopsy tissue cores and extensive proteomics and proteoforms to significantly obtain novel biomarkers to better predict HCC subtypes and their response to therapy.

Furthermore, by incorporating radiogenomics and radioproteomics, we aim to provide a comprehensive understanding of the molecular underpinnings of HCC across different radiological classifications, thus contributing valuable insights for clinical practice and research endeavors.

## Supplementary Material

Supplementary

## Figures and Tables

**Table 1.** The significantly upregulated genes > 2 fold, in moderate vs well differentiated tissue cores in both FF and FFPE, with greater fold change in FF, due to higher gene counts

	Frozen tissues (FF)			FFPE tissues		
Gene name	Normalized Gene Count moderate	Normalized Gene Count well	Fold-Change (moderate vs well)	Normalized Gene Count moderate	Normalized Gene Count well	Fold-Change (moderate vs well)
SCD	3280.54	101.16	32.41	1503.52	535.03	2.81
ACSL4	929.61	7.07	131.55	181.51	32.86	5.52
MBL2	392.09	15.94	24.58	194.96	65.80	2.96
RELN	596.45	24.03	24.81	293.06	81.29	3.61
GLUL	8238.2	300.55	27.41	4000.74	840.19	4.76

**Table 2.** Some of the upregulated proteins >1 fold, in (poor vs moderate) and (poor vs well) differentiated of the frozen tissues FF

Protein name	fold-change	fold-change
	poor vs moderate	poor vs well
Aldehyde dehydrogenase	1.34	1.53
Profilin-1	1.01	1.23
Actin-related protein	1.15	1.11
Isoform of P0DMV9, Heat shock 70	1.12	1.12
Alpha-actinin-4	1.32	1.35
Isoform of P32754	1.10	1.72
Catalase	1.00	1.98
Adenosyl homocysteinase	1.07	1.04
Serine hydroxymethyl transferase	1.49	1.74
Apolipoprotein A-I	1.44	1.17
Glycine amidino transferase	1.43	2.9
Myosin-9	1.02	1.06
Protein disulfide-isomerase A6	1.07	1.13
Sulfotransferase 1A1	1.22	2.27
Endoplasmin	1.18	1.03
Protein disulfide-isomerase	1.01	0.93
Isoform of P06737, Alpha-1,4	1.13	1.41
